# Discriminating rapid eye movement sleep from wakefulness by analyzing high frequencies from single-channel EEG recordings in mice

**DOI:** 10.1038/s41598-023-36520-7

**Published:** 2023-06-13

**Authors:** Sadegh Rahimi, Amir Soleymankhani, Leesa Joyce, Pawel Matulewicz, Matthias Kreuzer, Thomas Fenzl, Meinrad Drexel

**Affiliations:** 1 Department of Pharmacology, Medical University of Innsbruck, Innsbruck, Austria; 2 Neuroscience and Neuroengineering Research Laboratory, Iran University of Science and Technology (IUST), Tehran, Iran; 3 Clinic for Anesthesiology and Intensive Care, School of Medicine, Technical University of Munich, 81675 Munich, Germany

**Keywords:** Learning algorithms, Circadian rhythms and sleep, REM sleep

## Abstract

Rapid eye movement sleep (REMS) is characterized by the appearance of fast, desynchronized rhythms in the cortical electroencephalogram (EEG), similar to wakefulness. The low electromyogram (EMG) amplitude during REMS distinguishes it from wakefulness; therefore, recording EMG signal seems to be imperative for discriminating between the two states. The present study evaluated the high frequency components of the EEG signal from mice (80–500 Hz) to support REMS detection during sleep scoring without an EMG signal and found a strong positive correlation between waking and the average power of 80–120 Hz, 120–200 Hz, 200–350 Hz and 350–500 Hz. A highly negative correlation was observed with REMS. Furthermore, our machine learning approach demonstrated that simple EEG time-series features are enough to discriminate REMS from wakefulness with sensitivity of roughly 98 percent and specificity of around 92 percent. Interestingly, assessing only the higher frequency bands (200–350 Hz as well as 350–500 Hz) gives significantly greater predictive power than assessing only the lower end of the EEG frequency spectrum. This paper proposes an approach that can detect subtle changes in REMS reliably, and future unsupervised sleep-scoring approaches could greatly benefit from it.

## Introduction

Depending on the species, rapid eye movement sleep (REMS) accounts for 5–20% of adult mammalian sleep, and its detection and analysis are critical for the diagnosis of major sleep disorders^1^. REMS is characterized by rapid eye movements, cortical electroencephalogram (EEG) patterns characterized by high power of theta and desynchronized rhythms similar to wakefulness^2,3^, and muscle atonia in axial postural muscles^4^. Such remarkable similarities in temporal and spectral features in REMS and wakefulness challenge both automated and manually performed REMS detection.

Temporal and spectral EEG and electromyogram (EMG) features are currently used to manually score vigilance states in mice^5,6^. A number of studies have also proposed multi-channel machine learning methods to automate sleep scoring in rodents. They extract and transform multichannel EEG and EMG features to score REMS by using a K-nearest neighbors algorithm (k-NN), naive Bayes classifier (NBC), Convolutional neural networks (CNN), random forest, support vector machines (SVM), etc.^7^. For instance, Rempe et al. extracted features from standard frequency bands of the EEG power spectrum (below 30 Hz), added the root mean-squared EMG value and applied principal component analysis (PCA) and NBC. They achieved an accuracy of 57% and 70% to detect REMS based on 2 and 10 s epochs^8^. Using 23 frequency and time-domain features derived from EEG and EMG, Zeng et al. (2012) trained SVM and reached an overall positive predictive value and sensitivity of 72% and 62% to detect REMS^9^. After extracting time-invariant features from EEG and EMG data, Exarchos et al. applied a CNN-based method and achieved a mean accuracy of around 85–90% for REMS detection^10^. While EEG and EMG are commonly used for sleep scoring, apart from REMS, other stages can be determined from EEG only signals in humans^11^ and rodents^6^.

Due to the electroencephalographic similarities between REMS and wakefulness, the main current way to distinguish between these two vigilance states is the presence of muscle atonia in the EMG signal during REMS^12^. However, EEG and EMG equipment with multiple channels (without the application of sophisticated commutators, three-dimensional swivel systems, and carefully implanted EMG electrodes) can restrict a subject's movement and could interfere with sleep recordings due to discomfort. It could also save time during preparation and surgery by using a single-channel EEG for plain sleep scoring. Furthermore, some companies that provide telemetry EEG systems offer only one channel (e.g., Kaha Sciences). Thus, finding a quick and reliable way to discriminate REMS from wakefulness using single-channel EEG is reasonable. There are only two studies assessing REMS using single-channel EEG in mice. Liu et al. utilizes a multi-scale CNN to learn local time-invariant information^7^. They extracted global transition rules by a bidirectional attention-based temporal convolutional network and assessed datasets from three independent laboratories. They reported a macro-averaging F1 score of 69% to 86% for REMS between different cohorts of mice and rats. Tezuka et al. developed a real-time sleep stage classification system with a CNN, named the universal time-series network, processing raw EEG, FFT, and zeitgeber time together^13^ for 91% sensitivity and 98% specificity for REMS. Using supervised methods, both experiments reported very successful results; however, both assessed EEG frequencies up to 100 Hz and did not look at the possible role of higher frequencies in REMS detection, which would be affected by vigilance levels^14^ and could be used as a feature to score sleep stages in an entirely unsupervised manner.

Almost all studies in the domain of rodent sleep research apply the standard frequency bands δ, θ, α/μ, β and limit the spectrum of analysis to 30 Hz or maximum 100 Hz^15–18^. Despite the possibility to record with higher sampling rates, the use of a wider frequency range in detecting REMS has been totally neglected.

The present study evaluated the high frequency components of the EEG signal from mice (80–500 Hz) to support REMS detection during sleep scoring without an EMG signal. Based on this initial detection, a machine learning algorithm should be able to score REMS only on EEG features.

## Results

**Figure 1:**
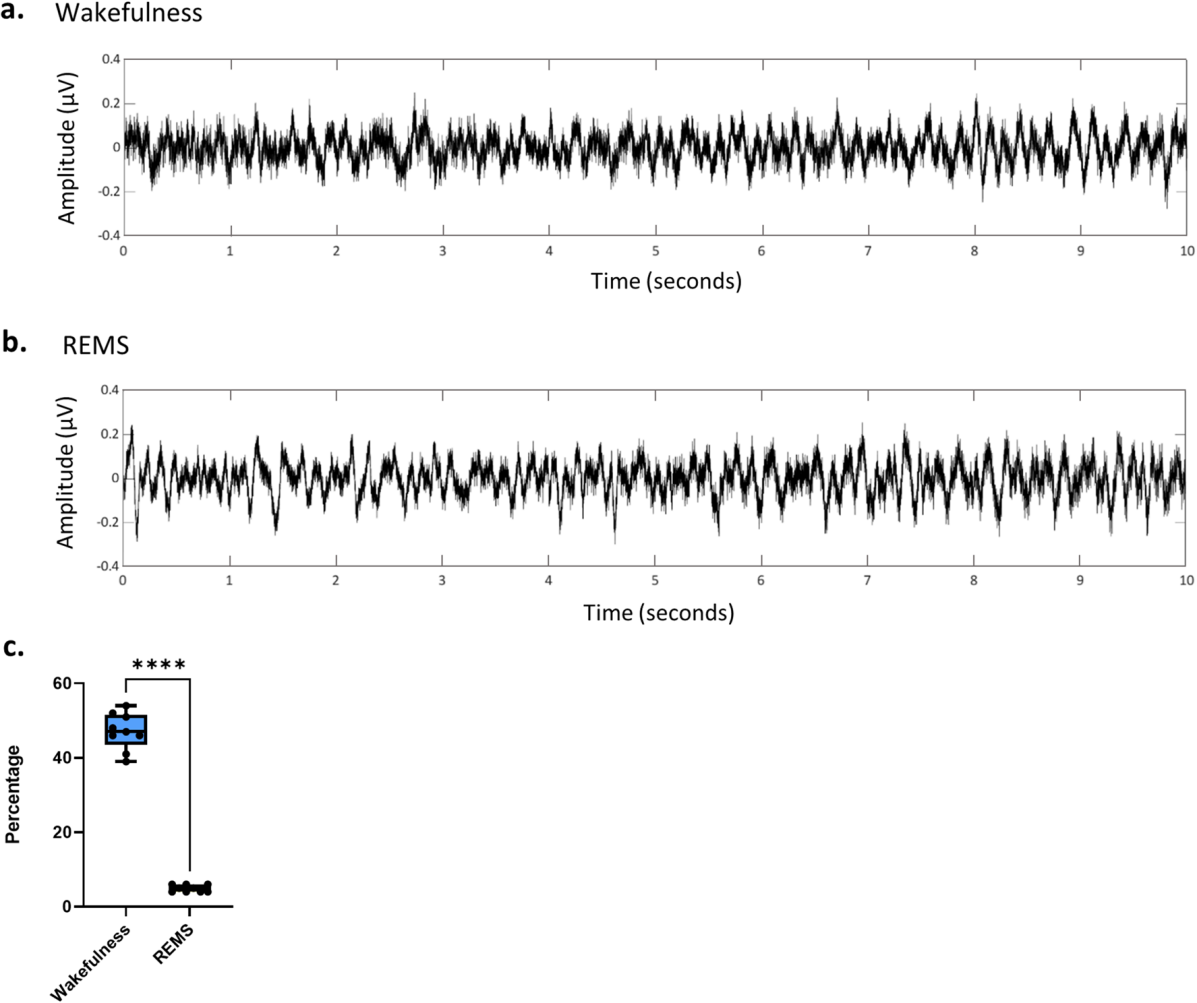
While EEG signals of wakefulness and REMS share some visual similarities, their imbalanced frequencies makes REMS detection difficult for machine learning. (**a**) A representative example of 10 s EEG trace of wakefulness (**b**) A representative example of 10 s EEG trace of REMS. (**c**) The median (range) percentage of wakefulness during 23 h of recording is 47 (15), while the value is 5 (2) for REMS according to manual scoring, showing significant difference (n = 9, *p* < 0.0001, according to Mann–Whitney unpaired test).

The median percentage of wakefulness was 47 (39 to 54), whereas REMS had a value of five (4 to 6). As expected, manually scored vigilance states by a sleep expert revealed a significant difference between the duration of wakefulness and REMS (*p* < 0.0001, Fig. 1, Supplementary Fig. S1 and Supplementary Fig. S2). A similar proportion has been reported in other studies in mice with the same genetic background^13^.

**Figure 2:**
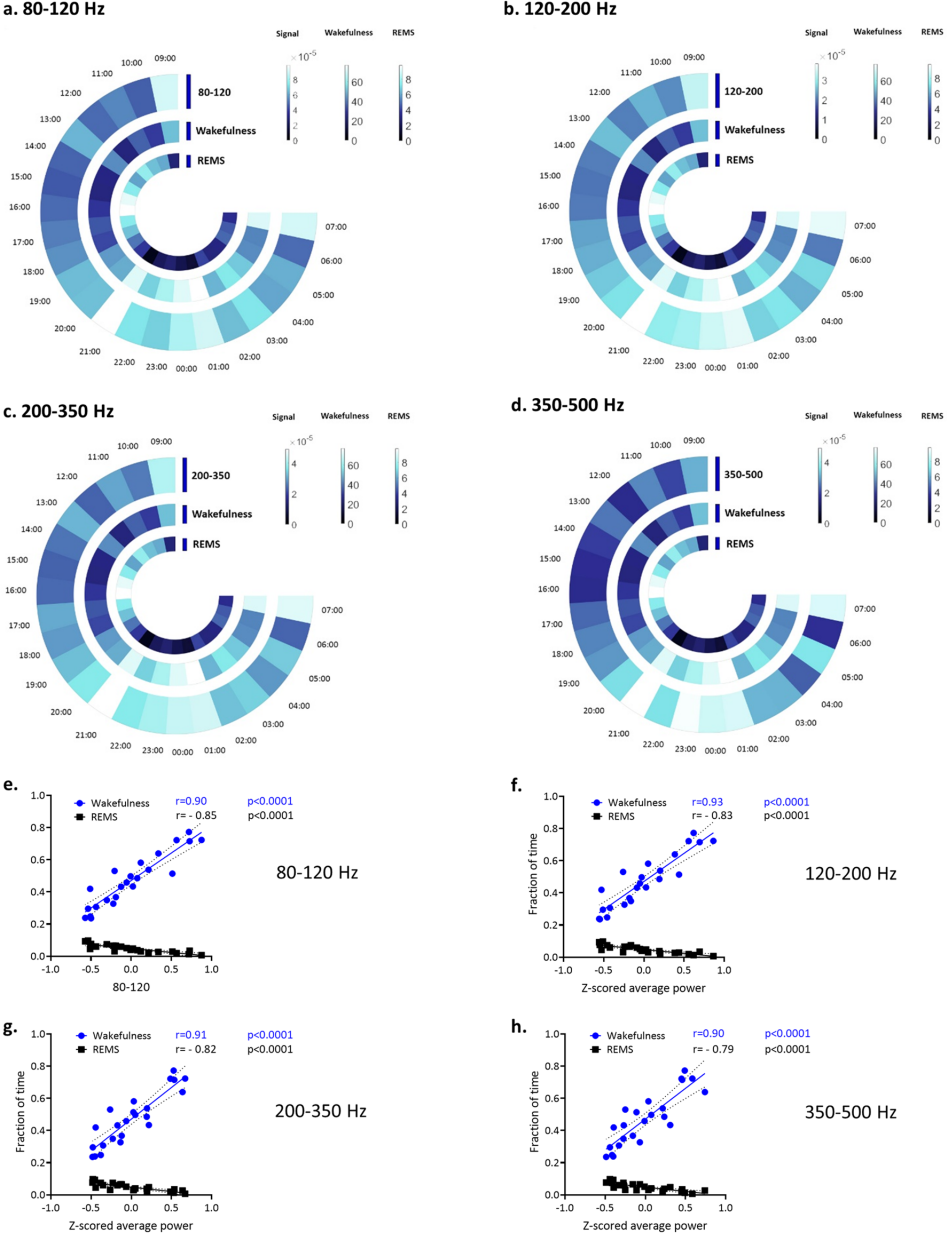
A comparison between z-scored-average power and mean percentage of wakefulness and REMS in each hour for different frequency bands. (**a**) to (**d**) represent the circadian rhythm of the z-scored-average power of 80–120 Hz, 120–200 Hz, 200–350 Hz and 350–500 Hz and the change in the mean percentage of wakefulness as well as REMS. Recording started at 09:00 and stopped at 08:00 the next morning. (**e**) to (**h**) show the significant positive correlation of wakefulness with the z-scored-average power of 80–120 Hz, 120–200 Hz, 200–350 Hz and 350–500 Hz, while REMS has a significant negative correlation with the value for each band (*p* < 0.0001, according to Pearson correlation coefficient test).

The Pearson correlation coefficient was measured to estimate the linear relationship between the z-scored-average power of 80–120 Hz, 120–200 Hz, 200–350 Hz, and 350–500 Hz with the percentage of wakefulness as well as REMS for each hour (Table 1). As is depicted in Fig. 2 (and Supplementary Fig. S3), during wakefulness, the z-scored-average power of 80–120 Hz, 120–200 Hz, 200–350 Hz and 350–500 Hz is significantly increased, while the value is decreased significantly for REMS (the power spectral density (PSD) was also evaluated, which shows the same trends, Supplementary Fig. S4). In mice, the waking is higher in the dark phase of the light–dark cycle while REMS is higher in the light phase of this nocturnal animal, which is interestingly correlated with the circadian rhythm of the z-scored-average power of 80–120 Hz, 120–200 Hz, 200–350 Hz, and 350–500 Hz (Fig. 2 a,b,c,d).

**Table 1:** Correlation of the hourly z-scored-average power of different frequency bands and the percentage of the mean REMS and wakefulness for the same hour.

	Pearson r	**80–120 Hz**	**120–200 Hz**	**200–350 Hz**	**350–500 Hz**
REMS	r	− 0.86	− 0.83	− 0.82	− 0.79
	95% Confidence Interval	− 0.94 to − 0.68	− 0.93 to − 0.64	− 0.92 to − 0.61	− 0.90 to − 0.56
	R squared	0.73	0.70	0.67	0.62
	P (two–tailed)	< 0.0001	< 0.0001	< 0.0001	< 0.0001

	Pearson r	**80–120 Hz**	**120–200 Hz**	**200–350 Hz**	**350–500 Hz**
Wakefulness	r	0.90	0.93	0.91	0.90
	95% Confidence Interval	0.78 to 0.96	0.83 to 0.97	0.80 to 0.96	0.77 to 0.96
	R squared	0.81	0.86	0.84	0.80
	P (two–tailed)	< 0.0001	< 0.0001	< 0.0001	< 0.0001

These findings led us to hypothesize that high frequencies from a single-channel EEG could be used to differentiate REMS from wakefulness; therefore, machine learning was applied to assess the possibility of distinguishing the two vigilance states. Furthermore, we sought to determine the relative contribution of each frequency band to REMS discrimination.

### Classification system

**Figure 3:**
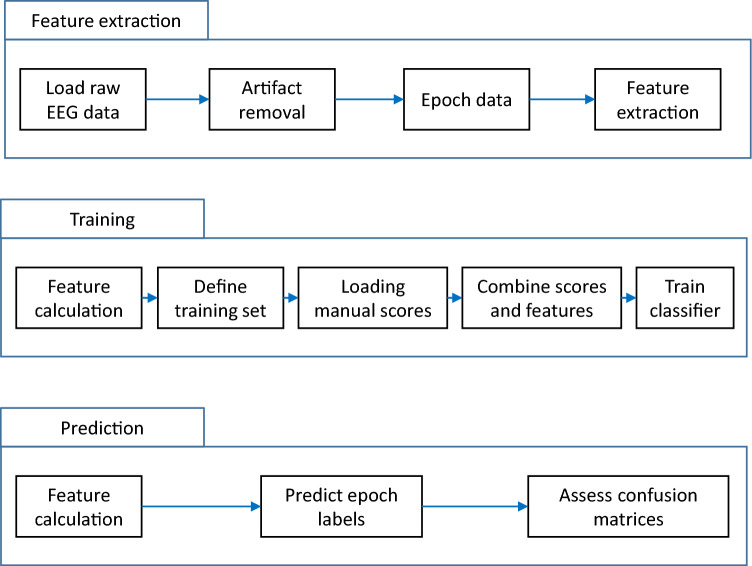
An overview of the network architecture used to distinguish REMS from wakefulness.

**Figure 4:**
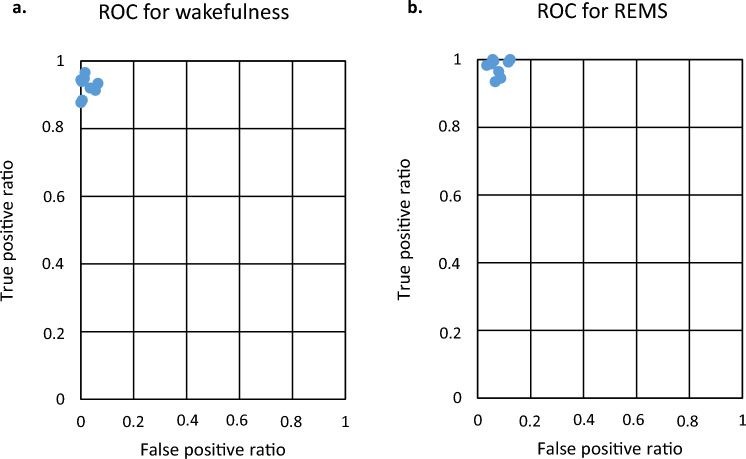
Depiction of prediction accuracy for each individual recording. The true and false positive ratios for each single animal (blue dots in the plot) are calculated based on the prediction of wakefulness (**a**) and REMS (**b**). Area under the curve is 0.98 ± 0.004.

**Figure 5:**
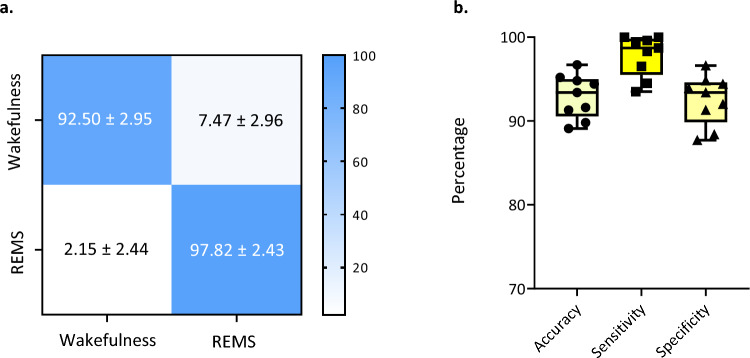
The algorithm's performance in discriminating between REMS and wakefulness (**a**) The confusion matrix. The percentage indicates the sample number of the predicted label divided by the total sample number of the corresponding true labels. (**b**) Boxplot of classification accuracy, sensitivity and specificity (each dot represents one recording).

Figure 3 shows the flow chart of the algorithm. Based on the features extracted from single-channel EEG recordings, NBC could distinguish REMS from wakefulness reliably and reached a mean ± SD accuracy, sensitivity, and specificity of 92.79 ± 2.75%, 98.20 ± 2.26% and 92.16 ± 3.18%, respectively. NBC was fed by features extracted from visual cortex recordings; however, it was also capable of discriminating REMS from wakefulness using the same features extracted from the sensory and motor cortex (Supplementary Table S1); therefore, the electrode location did not affect the algorithm's performance. True positive ratio (TPR) and false positive ratio (FPR) in predicting wakefulness and REMS were estimated. As presented in Fig. 4 as a receiver operating characteristic plot (ROC plot), the algorithm reached very high prediction accuracy for wakefulness as well as REMS. The mean ± SD of TPR and FPR for wakefulness were 0.92 ± 0.02 and 0.02 ± 0.02, respectively, compared to 0.97 ± 0.02 and 0.07 ± 0.02 for REMS (Area under the curve was 0.98 ± 0.004). The confusion matrix in Fig. 5a. summarizes the performance of the classification algorithm. To distinguish REMS from wakefulness, our kernel-based NBC model achieved accuracy, sensitivity, and specificity of 92.92 ± 2.60%, 97.82 ± 2.43% and 92.50 ± 2.95%, respectively (Fig. 5b and Table 2). To further validate the classifier, we compared it to the quadratic discriminant analysis (QDA), k-NN and SVM classifiers. While the kernel-based NBC model was superior to other models (Supplementary Table S2), three other models also predicted REMS very well, indicating that features are highly predictive in REMS discrimination.

**Table 2:** Accuracy, sensitivity and specificity of REMS prediction using kernel-based NBC (naïve Bayes classifier).

	Accuracy (%)	Sensitivity (%)	Specificity (%)
Minimum	88.80	94.40	87.40
Maximum	96.80	100	96.60
Mean	92.79	98.20	92.16
SD	2.75	2.26	3.18

**Figure 6:**
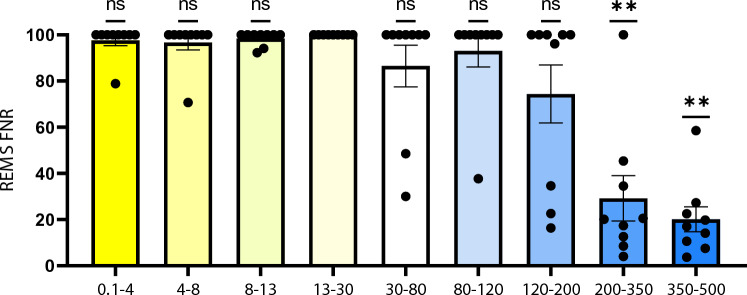
Assessing the level of predictability of each feature for separating classes. By limiting the algorithm to use only one frequency band, FNR (false negative ratio) in REMS prediction increased to almost 100 percent for 0.1–4 Hz, while it decreased significantly by choosing 200–350 Hz and 350–500 Hz (*p* < 0.01, according to one-sample Wilcoxon signed-rank test, compared with the hypothetical median of 100).

The algorithm was then fed with the average power of different frequency bands one by one and assessed the false negative ratio (FNR) of the REMS detection. Hence, it will reveal the frequency band(s) with a higher prediction value. As the proportion of REMS versus wakefulness is imbalanced (Fig. 1), assessing the FNR would indicate the cases where the algorithm neglected REMS and falsely chose wakefulness. As demonstrated in Fig. 6, if the algorithm is fed with only the average power of 0.1 to 4 Hz, the FNR will increase to nearly 100% (Supplementary Table S3) which means all epochs were considered wakefulness. This is also true for the average power of 4–8 Hz, 8–13 Hz and 13–30 Hz. Interestingly, the FNR decreased significantly by assessing only the average power of 200–350 Hz as well as 350–500 Hz (*p* < 0.01, Fig. 6, Supplementary Table S4), suggesting a prominent role for these frequency bands in discriminating REMS and wakefulness (also NREMS and wakefulness, but not NREMS from REMS, Supplementary Table S3 and Supplementary Table S4, Supplementary Fig. S4).

## Discussion

It is generally accepted that sleep–wake characteristics are found within the lower frequency end of the EEG spectrum^15^. However, we proposed the potential of higher frequency bands (above 80 Hz) in the sole EEG signal for REMS detection and found a strong positive correlation between waking and the average power of 80–120 Hz, 120–200 Hz, 200–350 Hz and 350–500 Hz. A highly negative correlation was observed with REMS. Furthermore, our machine learning approach demonstrated that simple EEG time-series features are enough to discriminate REMS from wakefulness with sensitivity of roughly 98 percent and specificity of around 92 percent. Interestingly, assessing only the higher frequency bands gives significantly greater predictive power than assessing only the lower end of the EEG frequency spectrum. To further validate our findings, we also assessed the accuracy, sensitivity and specificity of REMS discrimination with different classification models and electrode locations. Finding high prediction values supported the robustness of the features in REMS discrimination from wakefulness.

REM sleep is characterized by low EMG amplitude due to atonia in axial muscles, which is the main feature to distinguish REMS from wakefulness^4^; therefore, recording EMG signals seems to be imperative for discriminating between the two states. However, we reached high sensitivity and specificity in distinguishing REMS from wakefulness based on the features extracted from single-channel EEG, which in the context of the 3Rs (Replacement, Reduction, and Refinement) would mean considerable refinement. Eliminating EMG results in less pain for the animal and a lower risk of infection due to a simpler EEG montage. Furthermore, several research groups (particularly those in the field of epilepsy) already have extensive EEG databases, and with our EEG-only approach to REMS detection, they would all be able to utilize and assess the database from the point of change in sleep behavior, resulting in a reduction in the number of animals used for future experiments.

To our knowledge, there is no other study available using single or multichannel EEG, with or without EMG, to examine the possibility of scoring REMS using higher frequency bands. At present, sleep scoring tends to focus on the lower end of the EEG frequency spectrum. However, two studies assessed an extended range (above 200 Hz) of EEG activity across different vigilance states. Moffett et al., measured EEG activity in healthy, spontaneously behaving rats in the range of 200 to 2000 Hz^14^. The authors defined two new high-frequency EEG bands: Psi (ψ) centered between 260–280 Hz and omega (ω) with a broad peak in the 400–500 Hz range. In accordance with our study, the authors also found the values of the power spectral densities sum in ψ as well as ω band increased during periods of wakefulness. Another study by González et al. analyzed broad-band epidural EEG of rats using Permutation Entropy (PeEn) analysis up to 512 Hz^19^, and found that PeEn was maximal during wakefulness and decreased during sleep (REMS and NREMS). Similar to our findings, the authors reported the largest difference between wakefulness and REMS at the higher range of frequencies (above 200 Hz), which may support the utility of high frequency components of EEG in distinguishing between wakefulness and REMS.

Although high-frequency oscillations (ripples defined between 80–250 Hz and fast ripples between 250–500 Hz) have been extensively studied in epileptic rodents^20,21^, their physiological role is not well understood. Jones et al. used simultaneous in vivo intracellular and epipial field-potential recording to investigate the cellular correlates of fast oscillations in somatosensory cortex of healthy rats, evoked by vibrissa stimulation, and observed "fast oscillations" (200–400 Hz) and "very fast oscillations" (400–600 Hz) in the barrel cortex^22^. Additionally, EEG activity in the range of 200 Hz or higher has been observed in healthy rats following high-frequency stimulation of the thalamus^23^. Rodents' neocortex shows oscillations of 200–600 Hz (but not the hippocampus and parahippocampus)^24^, suggesting rapid integration of tactile information^25^. It is suggested that these oscillations may represent very fast interactions of local circuits established by cortical pyramidal cells during sleep and wakefulness^26^. We also observed a significant increase in the average power of higher frequencies during wakefulness, when animals are exposed to more tactile stimuli, which aligns with the proposed function of higher frequencies in integrating tactile information. However, additional research is necessary to fully elucidate the functional role of high-frequency oscillations in relation to vigilance states.

We assessed epidural EEG from motor, sensory and visual cortex and found no difference in the accuracy, sensitivity and specificity of the classifier. The findings indicated no significant differences in these parameters based on the location of the electrode, suggesting that the micro-structure motif and the dynamical behavior of the cortical signal were independent of the origin of the signal. Our results were consistent with a previous study by González et al., which also found no differences in PeEn analysis of vigilance states based on electrode location^19^. Interestingly, when comparing amplitude entropy values during REMS and wakefulness, the maximum differences were reported in the visual cortex (right and left hemisphere) compared to the motor and sensory cortex (right and left hemisphere). This finding is compatible with our slightly better results when the algorithm was fed by the visual cortex compared to the motor and sensory cortex. Our findings were also consistent with another study by Mondino et al., which assessed the spectral power and coherence of epidural EEG during different vigilance states in various cortical regions, including the olfactory bulb (OB), motor, sensory, and visual cortex (up to 200 Hz)^27^. The authors found differences between wakefulness and REMS in the higher frequency range more prominent than the lower frequency range of the EEG in all recording sites. Moreover, the authors reported a narrow peak in high-frequency oscillation at approximately 130 Hz that could only be observed during REMS in the OB and sensory cortices. This finding might explain our slightly lower accuracy and specificity in the sensory cortex compared to the motor and visual cortex. Overall, our results suggest that changes in high frequency oscillations during REMS and wakefulness are not significantly influenced by the recording site. However, further investigations are needed to fully understand this phenomenon.

One of the main distinctions between wakefulness and sleep is the absence of muscle tone and movement during sleep, particularly in REMS^4^. Muscle activity generates high amplitude, broad-spectrum electrical signals that overlap with the spectral range of neural high-frequency oscillatory activity, and several studies (reviewed here^28^) suggest that muscular activity could significantly contribute to surface EEG recordings in the high-frequency bands. To decrease contamination with the EMG artifact, skin was opened, holes were drilled and electrodes were inserted on the dura (see “Methods”). It was reported that the epidural recording is less vulnerable to EMG artifact^29–31^ and can significantly increase the ratio of ongoing brain activity to artifact amplitude in comparison of the simultaneously non-invasive EEG^32^. Additionally, a study by Hansen et al.^33^ found no significant differences in the trends of high-frequency oscillation (up to 200 Hz) changes between epidural recording and local field potential (LFP) recording. Since LFP is extracted from a comparatively small area of neuronal tissue located directly at the recording site, where the neuronal signal is generated, it is highly resistant to external muscle activity. This suggests that the signal-to-noise ratio of epidural recording is dependable even in high frequencies. Although we removed epochs with movement artifacts in our analysis, it is still possible that muscle activity contamination (mainly from muscle tone) reached the epidural electrodes through volume conduction, which could contribute to the recorded signal. Furthermore, we did not distinguish between immobile and mobile wakefulness, which have different EMG signature, adding to the complexity of the issue. More research is required to quantify the influence of muscle artifact during both active and inactive wakefulness, as well as sleep, on epidural recordings at high frequencies.

The application of machine learning to imbalanced databases is subject to difficulties, which is the main limitation of this study. The REMS periods are usually short and only around 5% of the entire day, which in the context of machine learning, can make the detection challenging. In this scenario, the classifier may have overclassified the majority class (wakefulness) and ignored the infrequent one (REMS), yet still reported a high level of accuracy (see “Methods”). To tackle the issue, instead of oversampling of REMS epochs, we assessed the raw values and reported the sensitivity and specificity of REMS discrimination, which are reliable representations of the classifier's performance. However, further research applying other techniques, in particular, the attention-based model^7^ can focus on preventing class-imbalance problems on a highly class-imbalanced dataset.

In the present study, we did not seek to present the best-performing REMS detection algorithm, but to introduce and evaluate a novel EEG-based feature in sleep scoring, which would improve REMS detection. Our approach is capable of detecting subtle REMS alternations reliably in fields where REMS detection is crucial (for instance, in psychiatric^34^ or degenerative diseases^35^). Future algorithms, in particular unsupervised techniques, could benefit greatly from this approach.

## Methods

### Animals

Nine adult (12–18 weeks, BW: 24–27 g) male wild-type mice (C57BL/6N, Charles River Laboratories GmbH, Germany) were used for this study. The mice were housed individually inside a custom-made, sound-attenuated recording box Faraday Chamber (bench-top Faraday cage, TMC, Peabody, MA, USA) under a 12/12-h light/dark cycle (lights ON: 9 am/lights OFF: 9 pm) and a room temperature of 22 ± 1 °C with a humidity of 50 ± 5% (food and water ad libitum). All experimental procedures were approved by the Committee of Animal Health and Care of the State of Upper Bavaria, Germany (ROB-55.2–2532.Vet_02–19–121) and conducted in accordance with relevant guidelines and regulations. Reporting in the manuscript follows the recommendations in the ARRIVE guidelines.

### Surgical procedure

The mice were anesthetized with 1.9–2.2% isoflurane (airflow rate 190–200 ml/min; Univentor 410 Anesthesia Unit, AgnTho's, Lidingö, Sweden) and were fixed in a stereotactic frame. The body temperature was maintained at 37 °C using a feedback-controlled heating pad (CMA 450, Harvard Apparatus, USA). For analgesia, Carprofen (0.004 mg/g of body weight) was subcutaneously injected in the scalp. The head was shaved, the scalp was opened medially and the periosteum was removed. Seven holes were drilled into the skull with a dental precision drill (Typ 4811, KaVo, Biberach, Germany). A PCP-Socket (PRECI-DIP SA Series 861, Switzerland) containing all electrodes was secured laterally using cyanoacrylate (UHU GmbH & Co. KG, Baden, Germany) and dental cement (Paladur, Heraeus-Kulzer, Hanau, Germany). Self-tapping jeweler’s screws (Ø 1.2 × 2 mm; Paul Korth GmbH, Lüdenscheid, Germany) were inserted into the two medial holes to stabilize the implant. Electrodes were made of 24 K gold (diameter 150 µm; Haefner & Krullmann GmbH, Germany). The tip of the electrode is ball-shaped to avoid injury as well as to improve surface contact. Three epidural EEG electrodes were implanted unilaterally on the left hemisphere (coordinates in mm from the bregma): primary visual cortex (V1, posterior, − 2.80, lateral, − 2.41), the primary sensory cortex (S1, anterior 1.94, lateral − 2.78), and the primary motor cortex (M1, anterior 1.10, lateral − 1.66). The grounding (reference) electrode was implanted most lateral to S1. A monopolar EMG electrode was inserted into the left neck muscle (Supplementary Fig. S6). For further information regarding the surgical procedure and electrode design, please refer to Fulda et al.^36^ and Fritz et al.^37^. The animals received analgesic treatment (Carprofen, 0.067 mg/ml) through drinking water from the pre-surgical day throughout post-surgical day 4.

### Data acquisition

After 10 days of recovery, the PCP-socket mounted on the animal’s head was connected to a pre-amplifier (amplification factor: 1x, custom made, NPI electronics, Tamm, Germany) and a commutator (SL-10 slip-ring commutator, Dragonfly Research & Development, Ridgeley, WV, USA) through a flexible recording cable. The commutator was mounted on a swivel system (custom made, M. Streicher, Innsbruck, Austria) that neutralizes weight and allows free movement of the animal in all three dimensions. After 4 days of adaptation to the cable attachment, 23 h of chronic EEG/EMG recordings were acquired. All recordings began at 09:00 and ended at 08:00 the following morning. Each recording channel was individually amplified (DPA-2FL, NPI electronics, Tamm, Germany) with a gain of 1000 × and filtered with a high-pass filter at 0.1 Hz, low-pass filter at 1000 Hz, and a 50 Hz hardware notch filter. The EEG and EMG signals were digitized with an analog–digital converter (Power1401-3A, Cambridge Electronic Design Limited, Cambridge, England) at a sampling frequency of 1000 Hz. All data were recorded with Spike2 Software (Version 7, CED, Cambridge, Great Britain, https://ced.co.uk/products/spkovin) and stored offline for further data analyses.

### Manual vigilance state classification

23 h of EEG/EMG recordings from each animal were divided into a light phase (first 12 h) and a dark phase (following 11 h). A semi-automated sleep scoring software^5,6,18^ was used to annotate vigilance states (Wake, NREMS, REMS) to every epoch (4 s long non-overlapping EEG episodes). The scoring software relies on manual thresholds to determine the sleep stages based on EMG- root mean squared (EMG RMS), delta and theta activities. NREMS is identified by low EMG RMS and high delta activity. A below threshold EMG RMS accompanied by below threshold delta activity and above threshold theta activity in the EEG signal was referred to REMS. Wakefulness was classified by high EMG RMS and low delta activity. The semi-automated sleep scores were manually reviewed and rescored considering a vigilant state that lasted at least 3 epochs as a behaviorally relevant state. Vigilance states lasting for less than 3 epochs were regarded as micro-states and were not considered as a vigilance state change. During the step for the manual confirmation, special care was taken to correctly classify epochs of quiet wakefulness based on the EEG features. Manually scored stages were based on the raw multichannel EEG and EMG; however, the EMG signal was dismissed for the further steps of the study and only the EEG was used. Artifact free EEG episodes were included in the study (the exclusion criteria were noisy EEG signals, e.g., more than 5% movement artifacts).

### Feature extraction

EEG data was segmented into consecutive 4-s, non-overlapping epochs that correspond to manually scored epochs. As sharp waveforms with asymmetric rising and falling phases can confound the spectral analysis^38^ the movement artifacts were removed before the FFT calculation. This was accomplished by manually defining a threshold and removing the peak of the artifacts, plus 50 ms before and after it. Then, features were extracted from the raw signal for all epochs. Features consist of the average power in the frequency range of 0.1–4 Hz, 4–8 Hz, 8–13 Hz, 13–30 Hz, 30–80 Hz, along with frequency ranges that are not commonly used in sleep studies, 80–120 Hz, 120–200 Hz, 200–350 Hz and 350–500 Hz. Outliers were detected using Grubbs’ test, which removes one outlier per iteration based on hypothesis testing and replaced with linear interpolation of neighboring, non-outlier values. Despite we recorded three EEG channels from the sensory cortex, motor cortex, and visual cortex, we analyzed each channel separately; therefore, the resulting feature vectors consisted of 9 elements (from 9 mentioned frequency bands). However, the study focused on less well-studied frequency bands above 80 Hz.

The percentage of the mean wakefulness and REMS for each one-hour period over 23 h was calculated. In order to make sure the results were valid across animals, the standard score (z-score) of each feature was calculated.

### Classification of the features

To distinguish REMS from wakefulness, kernel-based naïve Bayes classification (NBC) was employed. NBC is based on the hypothesis of conditionally independent predictors of the given classes. The dataset was randomly split into a training dataset containing 75% of the data points, which was used to train the classifiers based on the manual scoring, and a test dataset containing the remaining 25% of data points that were used to test the accuracy of the algorithms. In order to increase the reliability of the results, the dataset was randomly shuffled 1000 times and the algorithms were trained and tested on the respective datasets. The final classification result is reported based on the average of the 1000 times, however, each individual result was not qualitatively and quantitatively different from the final average. The figures are based on the analysis of features collected from the EEG of the visual cortex; however, the features from the sensory as well as motor cortex were also calculated and reported in the supplementary material. To determine if the features are sufficiently strong to produce similar predictive values in different classifiers, in addition to NBC, QDA, k-NN and SVM were assessed.

### Evaluation measures

The REMS and wakefulness scored by the expert occur with widely differing frequencies (Fig. 1), which can pose non-trivial challenges when creating data-driven systems due to the tendency of systems to over-classify majority class (wakefulness) and misclassify infrequent class (REMS)^39^. In this case, accuracy may not reflect actual performance, especially for a class with low frequency (i.e., REMS). Therefore, the results of the classification were reported by three indicators of accuracy, sensitivity, and specificity in REMS detection, which are defined as follows:

$$Accuracy = \frac{TP + TN}{{TP + FP + TN + FN}}*100\%$$

$$Sensitivity = \frac{TP}{{TP + FN}}*100\%$$

$$Specificity = \frac{TN}{{TN + FP}}*100\%$$

In the equations above, true positives (TP) is the number of epochs correctly scored as REMS, false positives (FP) is the number of epochs incorrectly scored as REMS, true negatives (TN) is the number of epochs correctly rejected as REMS, and false negatives (FN) is the number of epochs incorrectly rejected as REMS. For each time bin of 4 s, measurements were individually computed and the outcomes were presented as mean ± SD.

A ROC plot demonstrates our binary classifier system's diagnostic ability for individual recordings based on the TPR and FPR. To summarize the prediction performance of all 9 recordings, we used confusion matrix. Each row of the matrix represents the values in an actual class while each column represents the instances in a predicted class.

### Statistical analysis

Statistical analysis was performed using GraphPad Prism version 9.1 for Windows (GraphPad Software, USA, https://www.graphpad.com/). The Mann–Whitney U test was used to compare two datasets (wakefulness and REMS) that were not normally distributed. In order to assess the correlation between the average power of each frequency band and the percentage of wakefulness and REMS, despite applying a z-score to normalize data, normalization was further tested through the Shapiro–Wilk test. Afterward, the Pearson correlation coefficient was assessed. In order to determine which frequency band plays a pivotal role, a one-sample Wilcoxon signed-rank test to compare FNR in REMS detection for the power of each frequency band was applied. The hypothetical median was set to 100. Type I error was set at 0.05 for all tests.

## Supplementary Information

Supplementary Information.

## Data Availability

The scored EEG as well as EMG data, which were implemented in this work can be made available upon request by contacting the first author (Dr. Sadegh Rahimi, Medical University of Innsbruck, Austria, sadegh.rahimi@i-med.ac.at). The REMS discriminating algorithm is available at: https://github.com/rahimis851/Discriminating-REMS-from-wakefulness-by-analyzing-high-frequencies
