# Supplementary material for: Discriminating rapid eye movement sleep from wakefulness by analyzing high frequencies from single-channel EEG recordings in mice
Source: Sci Rep. 2023 Jun 13;13:9608. doi: 10.1038/s41598-023-36520-7 (PMC10264428; doi:10.1038/s41598-023-36520-7)
Supplement: Supplementary file 1 — Supplementary Information. [file 41598_2023_36520_MOESM1_ESM.docx]

**Supplementary Information**

Discriminating rapid eye movement sleep from wakefulness by analyzing high frequencies from single-channel EEG recordings in mice

Sadegh Rahimi^1§^, Amir Soleymankhani^2§^, Leesa Joyce^3^, Pawel Matulewicz^1^, Matthias Kreuzer^3^, Thomas Fenzl^3^, Meinrad Drexel^1^*

1. Department of Pharmacology, Medical University of Innsbruck, Innsbruck, Austria.
2. Neuroscience and Neuroengineering Research Laboratory, Iran University of Science and Technology (IUST), Tehran, Iran
3. Clinic for Anesthesiology and Intensive Care, School of Medicine, Technical University of Munich, 81675 Munich, Germany.

^§^ Authors contributed equally

* Corresponding author:

meinrad.drexel@i-med.ac.at

**Supplementary Table S1.** Accuracy, sensitivity, and specificity of REMS prediction by using recordings from the sensory and motor cortex (using NBC classifier). One-way ANOVA test did not find significant differences in the accuracy, sensitivity and specificity of REMS detection based on the electrode location.

| **Sensory cortex** | **Mean** | **SD** |
| --- | --- | --- |
| Accuracy (%) | 90.86 | 4.91 |
| Sensitivity (%) | 93.48 | 5.59 |
| Specificity (%) | 90.52 | 5.05 |

| **Motor cortex** | **Mean** | **SD** |
| --- | --- | --- |
| Accuracy (%) | 91.09 | 4.43 |
| Sensitivity (%) | 90.41 | 9.45 |
| Specificity (%) | 91.08 | 4.18 |

| **ANOVA summary** | **Accuracy** | **Sensitivity** | **Specificity** |
| --- | --- | --- | --- |
| F | 0.5811 | 3.298 | 0.3481 |
| P value | 0.5669 | 0.0543 | 0.7095 |
| P value summary | ns | ns | ns |
| R squared | 0.04619 | 0.2156 | 0.02819 |

**Supplementary** **Table S2.** Comparison of the different classifiers in accuracy, sensitivity, and specificity of REMS prediction. The values are expressed as a percentage (NBC= naïve Bayes classification, QDA= Quadratic Discriminant Analysis, k-NN= K-nearest neighbors algorithm, SVM= Support Vector Machine)

|  | **Mean** | **SD** |
| --- | --- | --- |
| **NBC**-Accuracy (%) | 92.92 | 2.60 |
| **NBC**-Sensitivity (%) | 97.82 | 2.43 |
| **NBC**-Specificity (%) | 92.50 | 2.95 |
| **QDA**-Accuracy (%) | 90.78 | 5.69 |
| **QDA**-Sensitivity (%) | 97.28 | 1.94 |
| **QDA**-Specificity (%) | 90.01 | 6.40 |
| **k-NN**-Accuracy (%) | 96.03 | 0.96 |
| **k-NN**-Sensitivity (%) | 77.85 | 5.77 |
| **k-NN-**Specificity (%) | 97.84 | 0.68 |
| **SVM**-Accuracy (%) | 95.77 | 2.61 |
| **SVM**-Sensitivity (%) | 74.90 | 18.65 |
| **SVM**-Specificity (%) | 98.16 | 0.78 |

**Supplementary Table S3.** The alteration of the FNR (false negative ratio) by feeding the algorithm with single feature

| REMS (vs wakefulness) | 0.1-4 Hz | 4-8 Hz | 8-13 Hz | 13-30 Hz | 30-80 Hz | 80-120 Hz | 120-200 Hz | 200-350 Hz | 350-500 Hz |
| --- | --- | --- | --- | --- | --- | --- | --- | --- | --- |
| Mean | 97.66 | 96.74 | 98.49 | 100.0 | 86.50 | 93.08 | 74.41 | 29.22 | 20.11 |
| SD | 7.033 | 9.767 | 3.032 | 0.000 | 27.18 | 20.77 | 37.71 | 29.45 | 16.19 |

| NREMS (vs REMS) | 0.1-4 Hz | 4-8 Hz | 8-13 Hz | 13-30 Hz | 30-80 Hz | 80-120 Hz | 120-200 Hz | 200-350 Hz | 350-500 Hz |
| --- | --- | --- | --- | --- | --- | --- | --- | --- | --- |
| Mean | 1.056 | 16.72 | 64.28 | 84.19 | 79.38 | 100.0 | 86.59 | 100.0 | 100.0 |
| SD | 0.7055 | 15.80 | 26.11 | 23.69 | 38.33 | 0.000 | 35.47 | 0.000 | 0.000 |

| NREMS (vs wakefulness) | 0.1-4 Hz | 4-8 Hz | 8-13 Hz | 13-30 Hz | 30-80 Hz | 80-120 Hz | 120-200 Hz | 200-350 Hz | 350-500 Hz |
| --- | --- | --- | --- | --- | --- | --- | --- | --- | --- |
| Mean | 33.50 | 36.62 | 50.96 | 57.71 | 81.64 | 58.38 | 59.29 | 26.97 | 11.04 |
| SD | 47.02 | 45.22 | 45.46 | 44.81 | 23.47 | 34.08 | 33.57 | 20.80 | 9.21 |

**Supplementary Table S4.** By feeding the algorithm with single feature, the median of FNR (false negative ratio) were compared in different frequency bands using one-sample Wilcoxon signed-rank test (with the hypothetical median of 100).

| REMS (vs wakefulness) | 0.1-4 Hz | 4-8 Hz | 8-13 Hz | 13-30 Hz | 30-80 Hz | 80-120 Hz | 120-200 Hz | 200-350 Hz | 350-500 Hz |
| --- | --- | --- | --- | --- | --- | --- | --- | --- | --- |
| Theoretical median | 100.0 | 100.0 | 100.0 | 100.0 | 100.0 | 100.0 | 100.0 | 100.0 | 100.0 |
| Actual median | 100.0 | 100.0 | 100.0 | 100.0 | 100.0 | 100.0 | 100.0 | 20.10 | 17.00 |
| Sum of signed ranks | -1.000 | -1.000 | -3.000 |  | -3.000 | -1.000 | -10.00 | -36.00 | -45.00 |
| Sum of positive ranks | 0.000 | 0.000 | 0.000 |  | 0.000 | 0.000 | 0.000 | 0.000 | 0.000 |
| Sum of negative ranks | -1.000 | -1.000 | -3.000 |  | -3.000 | -1.000 | -10.00 | -36.00 | -45.00 |
| P value (two tailed) | >0.9999 | >0.9999 | 0.5000 |  | 0.5000 | >0.9999 | 0.1250 | 0.0078 | 0.0039 |
| Significant? (alpha=0.05) | No | No | No |  | No | No | No | Yes | Yes |
| 95% confidence interval | 0.000 to 0.000 | 0.000 to 0.000 | -5.900 to 0.000 |  | -51.50 to 0.000 | 0.000 to 0.000 | -77.40 to 0.000 | -91.50 to -54.60 | -92.50 to -72.70 |

| NREMS (vs REMS) | 0.1-4 Hz | 4-8 Hz | 8-13 Hz | 13-30 Hz | 30-80 Hz | 80-120 Hz | 120-200 Hz | 200-350 Hz | 350-500 Hz |
| --- | --- | --- | --- | --- | --- | --- | --- | --- | --- |
| Theoretical median | 100.0 | 100.0 | 100.0 | 100.0 | 100.0 | 100.0 | 100.0 | 100.0 | 100.0 |
| Actual median | 1.000 | 16.28 | 61.17 | 92.05 | 100.0 | 100.0 | 100.0 | 100.0 | 100.0 |
| Sum of signed ranks | -45.00 | -45.00 | -45.00 | -28.00 | -3.000 |  | -1.000 | -45.00 | -45.00 |
| Sum of positive ranks | 0.000 | 0.000 | 0.000 | 0.000 | 0.000 |  | 0.000 | 0.000 | 0.000 |
| Sum of negative ranks | -45.00 | -45.00 | -45.00 | -28.00 | -3.000 |  | -1.000 | -45.00 | -45.00 |
| P value (two tailed) | 0.0039 | 0.0039 | 0.0039 | 0.0156 | 0.5000 |  | >0.9999 | 0.0039 | 0.0039 |
| Significant? (alpha=0.05) | Yes | Yes | Yes | Yes | No |  | No | Yes | Yes |

| NREMS (vs wakefulness) | 0.1-4 Hz | 4-8 Hz | 8-13 Hz | 13-30 Hz | 30-80 Hz | 80-120 Hz | 120-200 Hz | 200-350 Hz | 350-500 Hz |
| --- | --- | --- | --- | --- | --- | --- | --- | --- | --- |
| Theoretical median | 100.0 | 100.0 | 100.0 | 100.0 | 100.0 | 100.0 | 100.0 | 100.0 | 100.0 |
| Actual median | 8.454 | 67.56 | 90.19 | 91.22 | 60.38 | 66.31 | 14.40 | 7.985 | 8.454 |
| Sum of signed ranks | -28.00 | -28.00 | -21.00 | -36.00 | -45.00 | -45.00 | -45.00 | -45.00 | -28.00 |
| Sum of positive ranks | 0.000 | 0.000 | 0.000 | 0.000 | 0.000 | 0.000 | 0.000 | 0.000 | 0.000 |
| Sum of negative ranks | -28.00 | -28.00 | -21.00 | -36.00 | -45.00 | -45.00 | -45.00 | -45.00 | -28.00 |
| P value (two tailed) | 0.0156 | 0.0156 | 0.0313 | 0.0078 | 0.0039 | 0.0039 | 0.0039 | 0.0039 | 0.0156 |
| Significant? (alpha=0.05) | Yes | Yes | Yes | Yes | Yes | Yes | Yes | Yes | Yes |

**Supplementary Figure S1.** Individual sleep architectures. Waffle charts **(a)** to **(i)** present the proportion of wakefulness, REMS and NREMS for each recording (each circle representing 1% of data)

Recording 1

Recording 2

Recording 3

Recording 4

Recording 5

Recording 6

Recording 7

Recording 8

Recording 9

**a.**

**b.**

**c.**

**d.**

**e.**

**f.**

**g.**

**h.**

**i.**

**Supplementary Figure S2.** The circadian rhythm of wakefulness **(a)**, REMS **(b)** and NREMS **(c)** recorded from 9 mice. Mice are nocturnal animals and sleep longer during the light phase.

**a.**

**b.**

**c.**

Light

Dark

Light

Dark

Light

Dark

**80-120 Hz**

Time (Hours)

Power (dB)

**Supplementary Figure S3.** An example of the average power of 80-120 Hz, 120-200 Hz, 200-350 Hz and 350-500 Hz from one recording, compared to the corresponding hypnogram. Yellow highlights pronounce epochs of wakefulness in the hypnogram and corresponding time in the frequency bands.


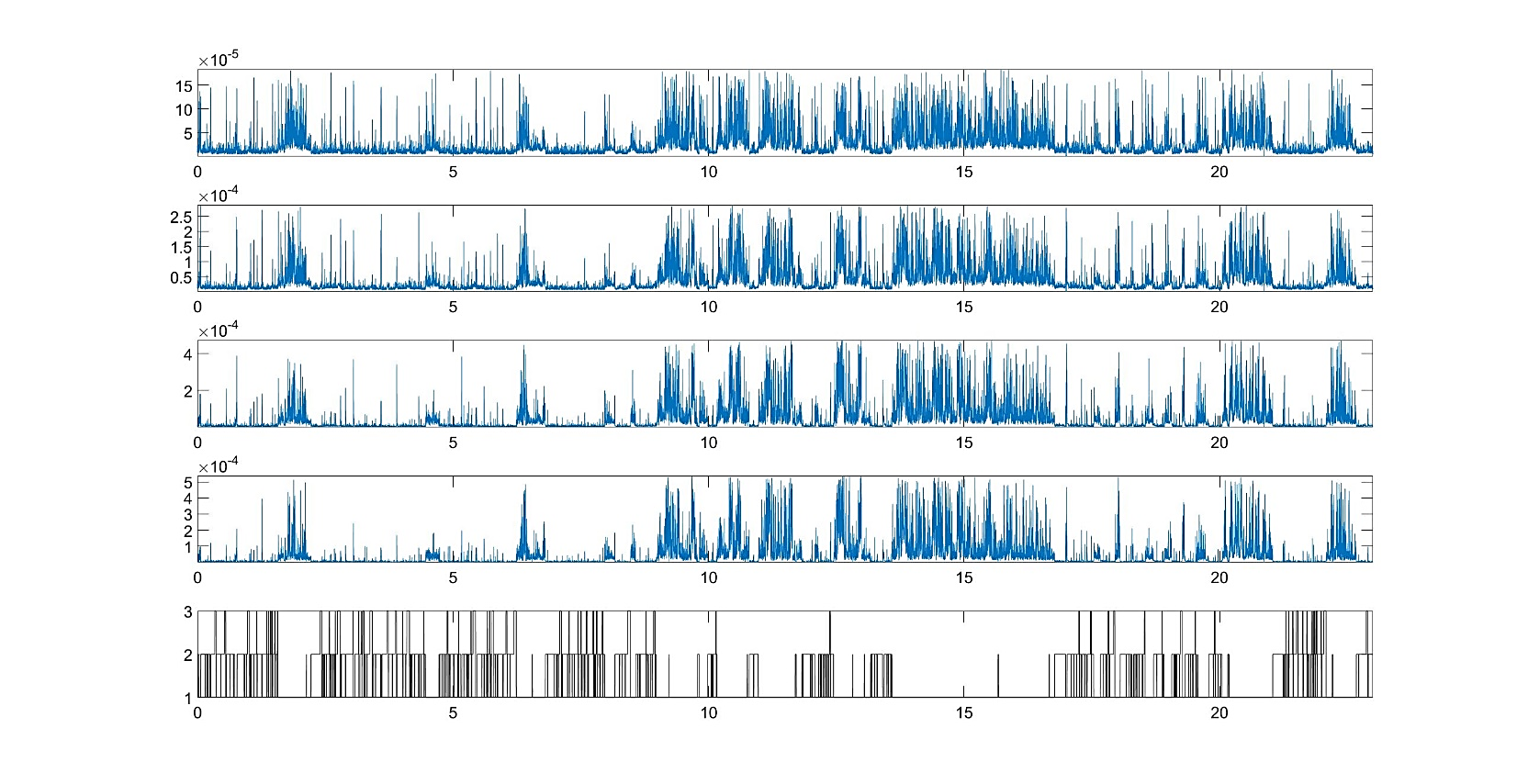

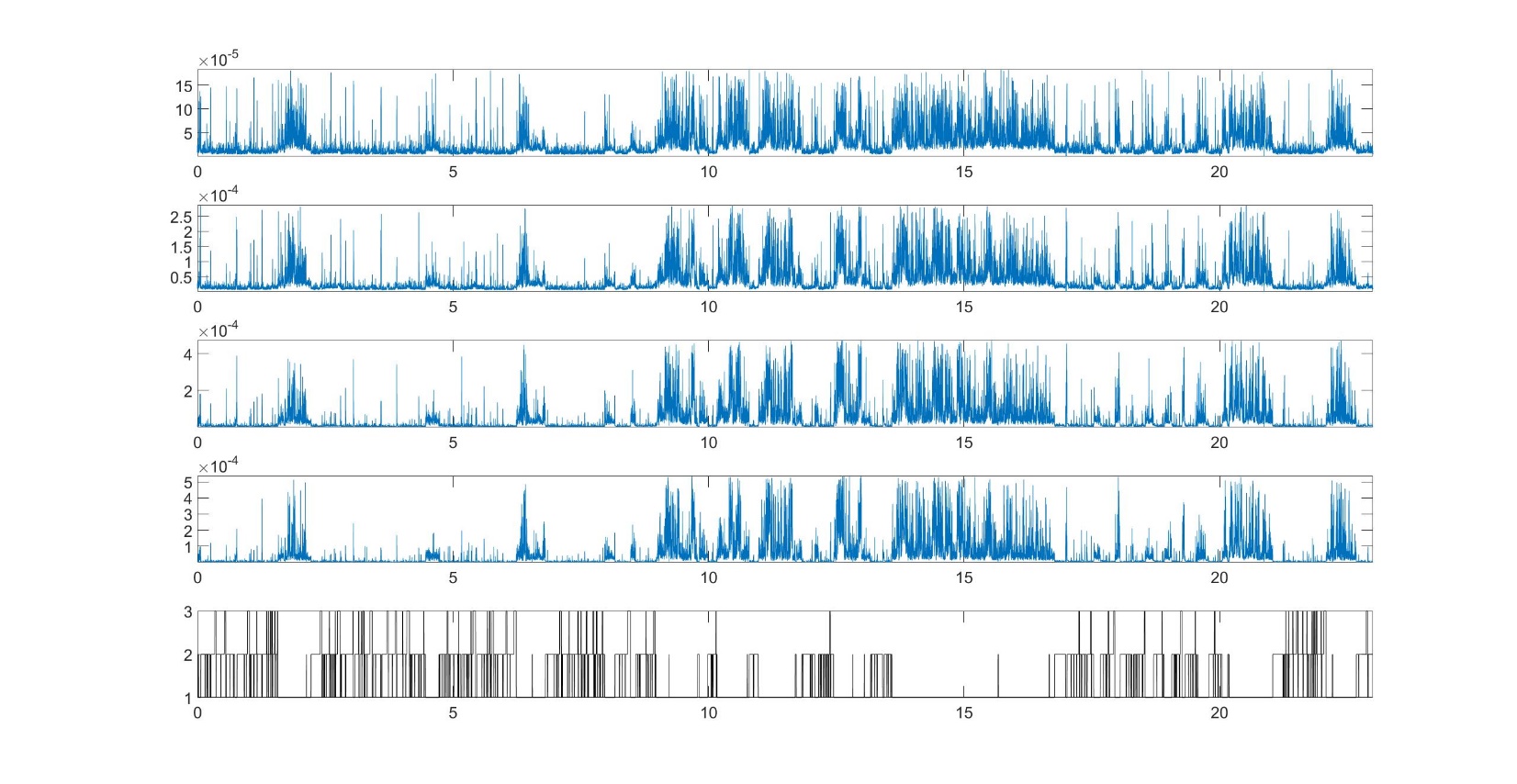

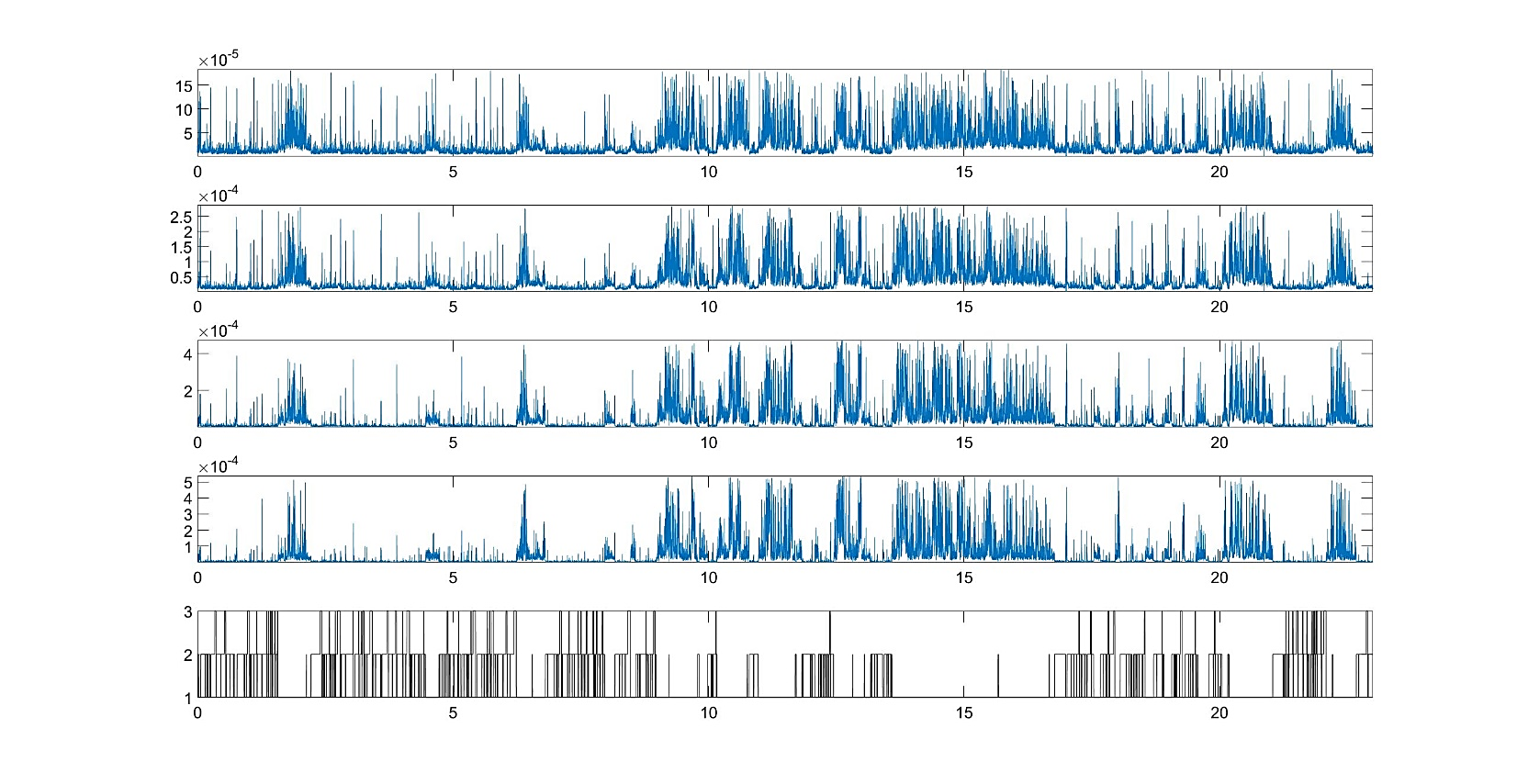


**120-200 Hz**

Time (Hours)

Power (dB)


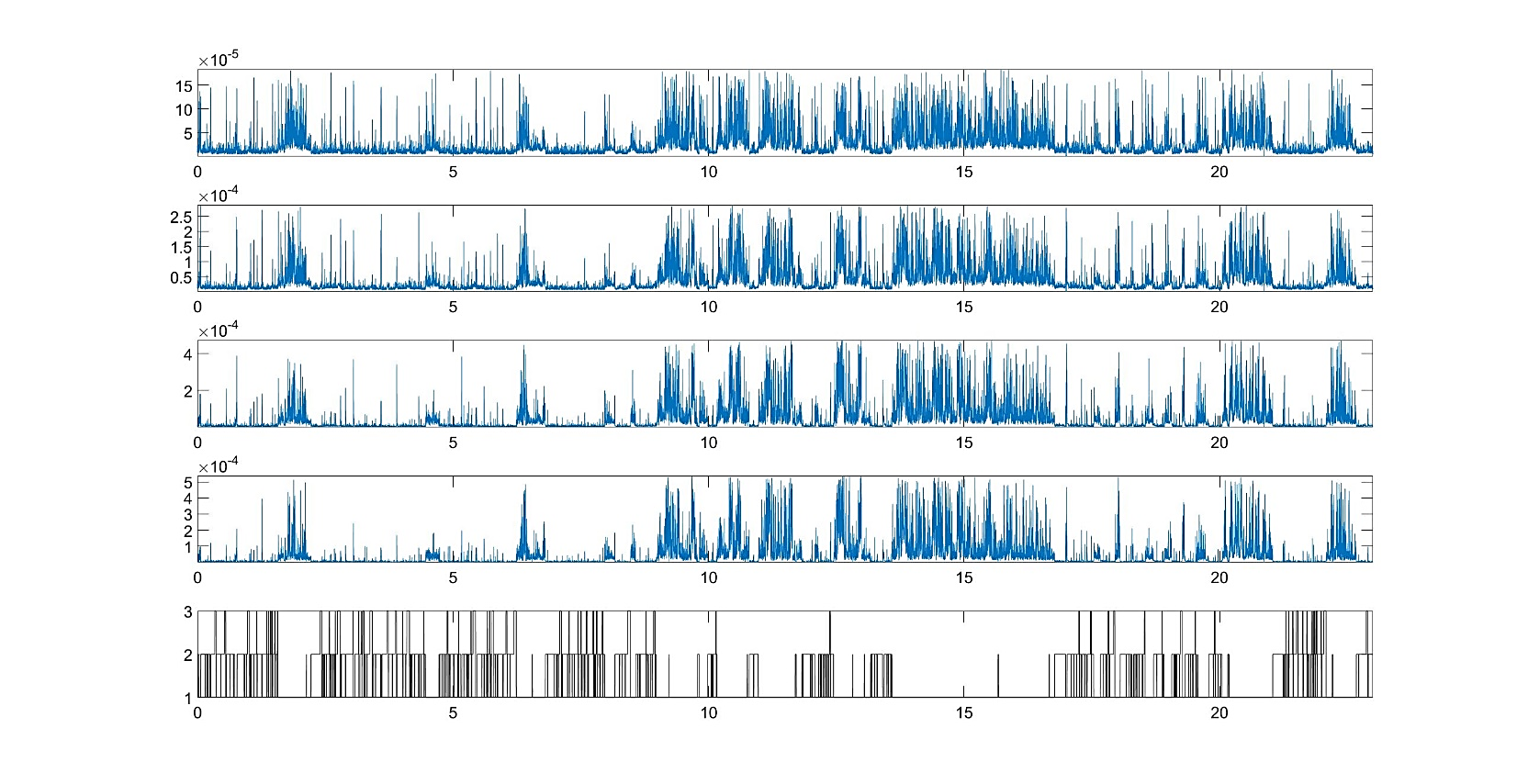


**200-350 Hz**

Time (Hours)

Power (dB)


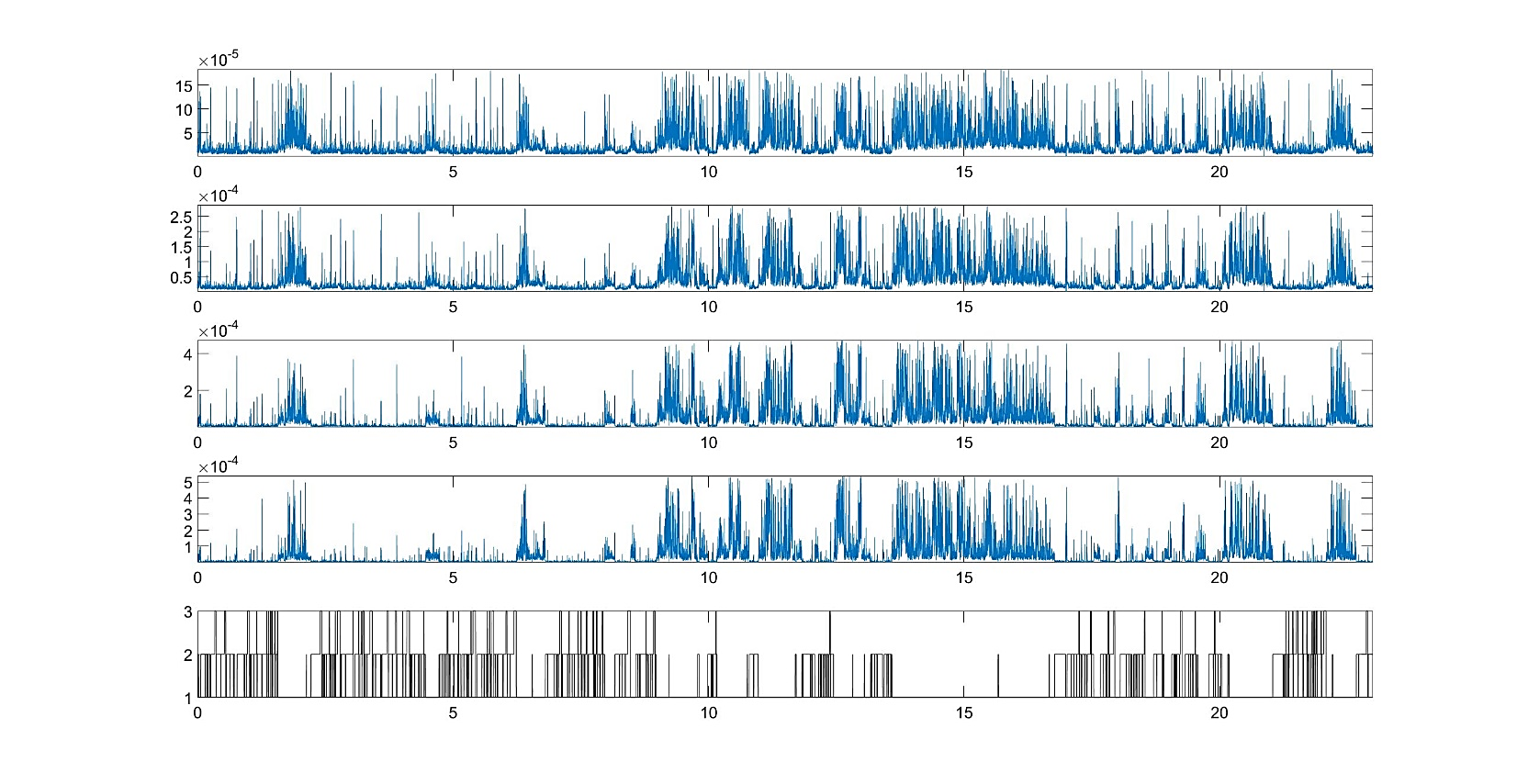


**350-500 Hz**

Time (Hours)

Power (dB)

Time (Hours)

**Hypnogram**

Wake

NREMS

REMS

**Supplementary Figure S4.** The power spectral density (PSD) profile of different vigilance states. Expanding the spectrum analysis up to 500 Hz facilitates the discrimination between wakefulness and REMS (the significant decrease in power observed in each 50 Hz frequency band is a result of implementing the notch filter).


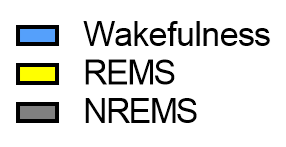


**Supplementary Figure S5.** Assessing the level of predictability of each feature for separating classes. By limiting the algorithm to use only one frequency band, FNR (false negative ratio) in class discrimination is assessed. High frequency oscillations are helpful in discriminating NREMS from wakefulness **(a)**, but not NREMS from REMS **(b)**

**a.**

**b.**


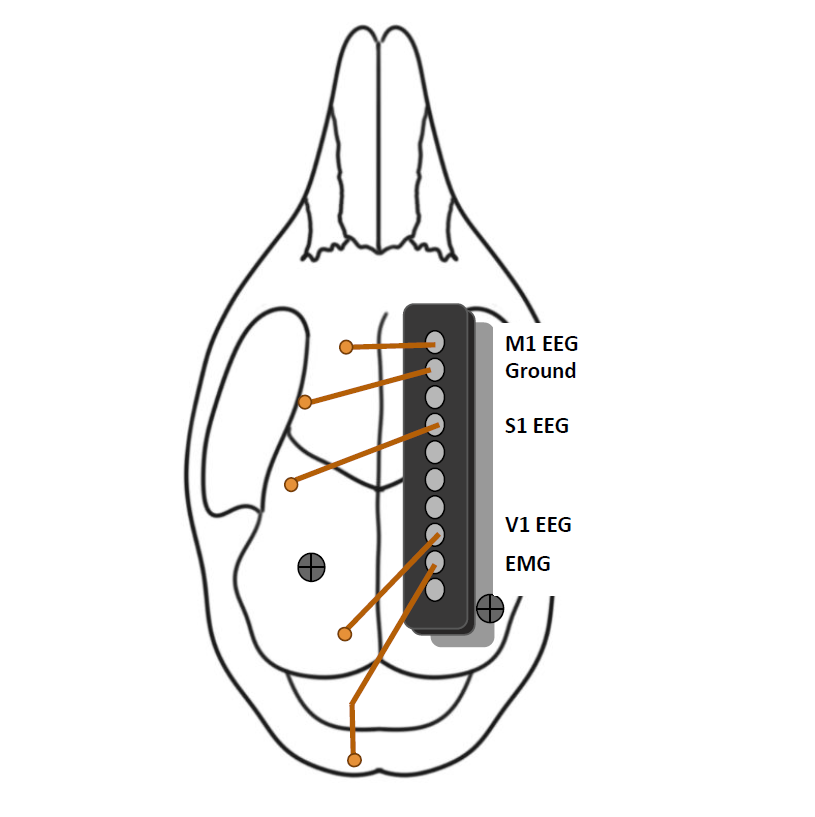


**Supplementary Figure S6.** Electrode design and implantation: EEG and EMG implantations on the mouse skull in the left hemisphere along with two anchor screws implanted for stability. M1: motor cortex, S1: sensory cortex, V1: visual cortex.
